# Investigation of the Effects of Magnesium-Sulfate as Slag Activator

**DOI:** 10.3390/ma13020305

**Published:** 2020-01-09

**Authors:** Choonghyun Kang, Taewan Kim

**Affiliations:** 1 Department of Ocean Civil Engineering, Gyeongsang National University, Tongyeong 53064, Korea; chkang@gnu.ac.kr; 2 Department of Civil Engineering, Pusan National University, Busan 46241, Korea

**Keywords:** alkali-activated slag, magnesium sulfate, ettringite, gypsum, pore structure

## Abstract

This study is about the mechanical and microstructural properties of alkali-activated slag (AAS) paste using magnesium sulfate (MS) as an activator. MS is 2%, 4%, 6%, 8% and 10% contents of binder weight and water-binder ratio is 0.35. Compressive strength, X-ray diffraction, mercury-intrusion porosimetry, and thermal analysis were performed for analysis. The MS contents at which the maximum compressive strength appeared varied according to the measurement age. Hydration products affecting compressive strength and pore structure were ettringite and gypsum. As a result, the changes of ettringite and gypsum depending on the contents of MS have a great influence on the pore structure, which causes the change of compressive strength. The high MS contents increases the amount of gypsum in the hydration products, and the excess gypsum causes high expansion, which increases the diameter and amount of pores, thereby reducing the compressive strength.

## 1. Introduction

Recently, eco-friendly cement has attracted much research interest. In particular, alkali-activated slag cement (AASC) is a blend of ground granulated blast-furnace slag (GGBFS) and activators [1,2]. Many factors affect the characteristics of AASC, including (i) the fineness and nature of the GGBFS, (ii) the nature of the activator, (iii) the dosage of activators and (iv) the curing temperature [3,4,5,6]. However, regarding the mechanical properties of AASC, one of the most important factors is the nature of the alkali activator. The activators used in AASC can be classified into solutions and solid powders [7]. In previous studies on AASC, sodium hydroxide (NaOH) [8,9], sodium silicate (Na_2_SiO_3_) [4,10,11] or sodium carbonate (Na_2_CO_3_) [5,6] has been used as the single activator, and some researchers have even used blends of these activators [3,7,8,9,10,11]. Some researchers have also reported that the silica modulus of sodium silicate influences the properties of alkali-activated slag [12].

In studies on ordinary Portland cement, Na_2_SO_4_ and magnesium sulfate (MgSO_4_, hereinafter denoted as MS) have been mainly used for sulfate immersion experiments [13]; these sulfates have been studied as important factors causing the deterioration of ordinary Portland cement. Meanwhile, degradation studies by sulfate immersion experiments have also been conducted on AASC [9,14], but some sulfates have also been used as activators in AASC. Sodium sulfate (Na_2_SO_4_) [15,16,17,18] or gypsum (CaSO_4_·2H_2_O) [19,20] was used as an activator in the AASC study using sulfate.

Mobasher et al. [15] studied how the sodium sulfate content and curing duration affect the binder structure of sodium-sulfate-activated slag cement; they prepared activating solutions containing 5, 10 and 25 wt% Na_2_SO_4_ by dissolving solid anhydrous Na_2_SO_4_ in distilled water. Rashad et al. [16] reported that the most significant changes upon curing at long-term ages observed were growth of the ettringite (3CaO·Al_2_O_3_·3CaSO_4_·32H_2_O) and an increase in silicate chain length in the C-A-S-H, resulting in higher strength. Mobasher et al. [17] studied composites of Ba(OH)_2_, Na_2_SO_4_ and blast-furnace slag (BFS) for immobilising sulfate-bearing nuclear waste. Rashad [18] reported the effect of five additives (silica fume, fly ash, limestone, hydrated lime and Portland cement) on the workability, compressive strength and drying shrinkage of 1% Na_2_O equivalent of Na_2_SO_4_ activated slag. Chang et al. [19] studied how gypsum (CaSO_4_·2H_2_O) and phosphoric acid affect the properties of sodium-silicate-based alkali-activated slag (AAS) paste. Neto et al. [20] reported the characteristics of BFS pastes activated with hydrated lime (5%) and hydrated lime (2%) plus gypsum (6%) in relation to compressive strength, shrinkage (autogenous and drying) and microstructure (porosity, hydrated products). Rashad et al. [21] investigated how the slag fineness and Na_2_SO_4_ dosage affect the strength, pH, hydration products and microstructure properties, compared with those of ordinary Portland cement. Finally, Park et al. [22] studied the strength and porosity of GGBFS activated by CaO with added gypsum. There are also attempts to develop new activators that are different from the ones applied by previous studies [23].

Unlike Na_2_SO_4_, MS is used very rarely as an activator. Previous AASC studies have shown that Na_2_SO_4_, CaSO_4_ or CaSO_4_·2H_2_O can be used as a sole activator at room temperature. However, data on sulfate activators other than these two are not sufficient. Especially, the sulfate activator has low activity in the early stage of hydration, so slow setting time and low initial strength are important disadvantages. Research is needed to develop new sulfate activators and to improve disadvantages of sulfate activators.

It is a process necessary to improve the performance of AASC through the research and development of various activators. The experiments conducted in this study aim to determine the possibility that MS can be used as an activator of AASC. From previous studies, Na_2_SO_4_, CaSO_4_ or CaSO_4_·2H_2_O is used as an activator of AASC but not MS. Therefore, experiments and analysis on the basic characteristics of AASC using MS as an activator are performed. This research focuses on investigating fundamental properties when MS is used as the sole activator of slag. In order to do so, we present data of compressive strength measurements, X-ray diffraction (XRD), thermal analysis and scanning electron microscopy (SEM) coupled with energy-dispersive X-ray spectroscopy (EDS).

## 2. Materials and Methods

### 2.1. Materials

The components of the GGBFS used in the experiments were identified using X-ray fluorescence analysis (the composition and analysis results are given in Table 1). The magnesium sulfate (MS) that was used (98% purity, anhydrous) came from SAMCHUN Chemicals (Seoul, Korea). Magnesium sulfate (MgSO_4_) is a white powder with a density of 2.7 g/cm^3^ and a pH value of 7.9 (50 g/L, H_2_O, 25 °C).

### 2.2. Experimental Procedures

A paste was prepared with a water-to-slag ratio of 0.35, as determined by performing preliminary experiments and measuring the appropriate fluidity and setting time. Regardless of the mixing ratio of MS, the flow values of all pastes were 180 ± 10 mm, which did not change much. MS was substituted for 2%, 4%, 6%, 8% or 10% of the mass of the mixing water. The 0% magnesium sulfate sample could not be tested because it was not setting and hardened. Therefore, 0% MS sample was excluded. The paste was prepared according to the ASTM C305 [24] standard. After the mixing was completed, the paste was poured into a 50 × 50 × 50 mm^3^ cube mould and placed in a chamber at a temperature of 80 ± 2 °C and relative humidity of 95 ± 5% for 24 h. From previous studies, sulfate-based activators showed low activation effects at room temperature. Therefore, some researchers cured at high temperature, not room temperature. High temperature curing had the effect of improving the slow setting time and low initial strength of the sulfate-based activator. Sahin et al. [25] selected a curing temperature of 80 °C in an experiment with gypsum. Rashad et al. [21] set the curing temperature to 40 °C in an experiment using Na_2_SO_4_ as an activator. This study also referred to the results of previous researchers. In preliminary experiments, setting and hardening of samples using MS was excessively delayed at room temperature. Therefore, the high temperature curing of 80 °C was chosen.

The moulds were then removed, and the sample was stored in another chamber at a temperature of 23 ± 2 °C and a relative humidity of 90 ± 5% until measurements were conducted.

The compressive strengths of the samples were measured according to the ASTM C109 [26] standard after 3, 7 and 28 days following preparation. All values are reported as the average of three samples. After the compressive strength was measured, the broken pieces were immersed in acetone for 12 h, dried in a vacuum desiccator for 24 h and then pulverised into fine particles and used for XRD (Xpert3; Malvern Panalytical, Malvern, UK). The analysis is from 5° to 60° (2θ), the step size is 0.017° (2θ), and Cu-K radiation (λ = 1.54 Å). For the analysis of hydration products, HighScore Plus software was used.

On the 28 days of hardening, the middle portion of the sample was cut into small cube pieces with a size of 5 mm, and mercury intrusion porosity (MIP) measurement (AutoPore IV 9500, Micromeritics Instrument Corp., Norcross, GA, USA) was performed. The sample was immersed in acetone for 12 h to stop hydration and then dried in a vacuum desiccator for 24 h to remove any residual acetone and moisture inside the sample. The MIP analysis was carried out with a mercury contact angle of 130°, a surface tension of 485 mN/m and a density of 13.534 g/mL for pore sizes of 0.003–336 μm. The measurement of pH was determined by the method performed in Rashad et al. [21]. For pH measurement, mix the mass ratio of powder:distilled water to 1:10 and stir for 24 h on a roller table. Next, measure the pH of the separated supernatant liquid after 5 min operation at 5000 rmp in a centrifuge.

Hydration products were observed by SEM (SUPRA 40; Zeiss, Oberkochen, Germany) using the crushed sample fragments from measuring the compressive strength. After the compressive strength was measured, the broken pieces were immersed in acetone for 12 h and then dried in a vacuum desiccator for 24 h. A representative secondary-electron image of the microstructure was taken in high-vacuum mode using an accelerating voltage of 15 kV and working distance of 9.8–10 mm. The samples with hydration stopped were platinum coated. The SEM with energy dispersive spectroscopy (EDS) was used to take secondary-electron images of the 28 days specimens. Thermal analysis (TG/DTG) was performed for 3-day and 28-day specimens. The TG/DTG was measured with a PerkinElmer DSC800 (Perkin Elmer, MA, USA), with a measurement temperature range of 30–800 °C and a temperature rise rate of 20 °C/min. The sample was N_2_ gas atmosphere.

## 3. Results and Discussion

### 3.1. Compressive Strength

**Figure 1:**
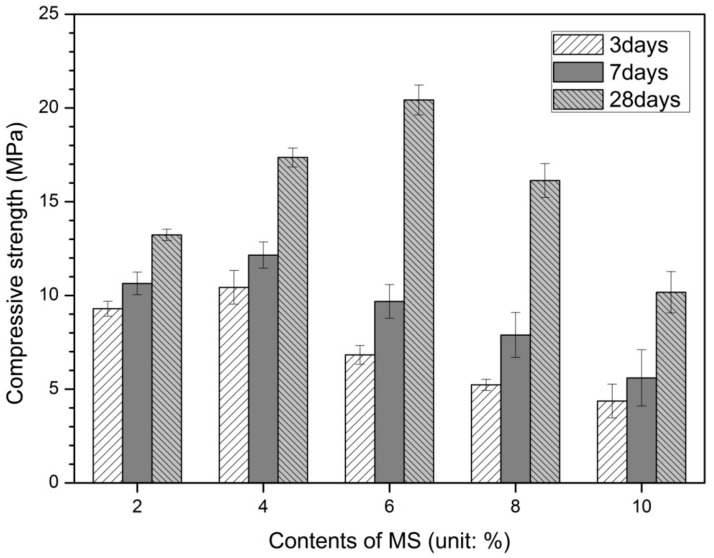
Compressive strength according to MS contents.

Figure 1 shows the measured compressive strength according to the MS concentration and age. Regardless of the amount of MS, the compressive strength increased with age from 3 to 28 days.

The highest values of compressive strength were found at a MS contents of 4% (10.43 MPa), 4% (12.15 MPa) and 6% (20.43 MPa) respectively for 3 days, 7 days and 28 days of curing, and then they decreased with the increasing of the MS contents, showing the lowest compressive strength value at 10% MS (in Figure 1). As shown in Figure 1, the highest compressive strengths at 3 days, 7 days, and 28 days were observed in the contents of a particular MS. These results indicate that the compressive strength does not increase linearly with increasing MS contents. The highest values of compressive strength increased with the age, for all the MS contents, as expected [3,5,18].

Rashad et al. [16] reported that the compressive strength of this study. Rashad et al. [16] performed sodium sulfate activation on two types of slag with different fineness of 2500 cm^2^/g and 5000 cm^2^/g. Sodium sulfate is 2.29% and 6.87% of the weight of slag, which means 1% and 3% of Na_2_O equivalent by mass of slag, and the water-to-binder ratio (w/b) is 0.3. The higher the fineness of the slag, the higher the compressive strength at the same sodium sulfate concentration. Except for the 3 days compressive strength of M2, the strengths of 3, 7 and 28 days of the samples were higher than the values of this study. As the concentration of sodium sulfate increased from 2.29% to 6.87%, the compressive strength increased regardless of slag fineness. However, this study shows low initial compressive strength despite increasing MS concentration. It also showed the highest compressive strength at a specific MS concentration. The fineness of slag used in this study is in the middle of the two kinds of slag fineness used in the Rashad et al. [16] experiment. However, regardless of the fineness characteristics, MS did not show a significant contribution to the improvement of compressive strength due to lower activation of slag than sodium sulfate.

Melo Neto et al. [20] reported the compressive strength results of mortar test using 2% lime + 6% gypsum on slag mass. The ratio of binder:quartz sand:water was 1:2:0.48. In the compressive strength of this study, the 3 and 7 days strengths were higher than all MS concentrations. However, the compressive strength of 28 days showed a slight difference than that of 6% MS. Park et al. [22] reported the results of replacing gypsum with 10% and 15% of the slag mass. All slag used in the experiment basically substituted 5% CaO. All mixtures have a constant w/b of 0.4. The 10% gypsum shows higher compressive strength than 15% concentration. In addition, 10% and 15% gypsum samples showed higher compressive strength than MS at 7 and 28 days. In the case of gypsum, there was a specific concentration with the highest compressive strength. In this study, the highest compressive strength occurred at 4% at 3 and 7 days and 6% MS at 28 days. Park et al. [22] reported that when the concentration of gypsum increased from 10% to 15%, the change in pore structure (increase of total porosity) due to the expansion of unhydrated gypsum was the cause of the decrease in strength.

Haha et al. [27] used NaOH as 3.77% of the slag mass and water glass as 10.0% to have the same Na_2_O content. Here, M8 means 7.7% of MgO contents, 10.5% of M11, and 13.2% of M13 among the components constituting slag. The w/b ratio is 0.4 with paste. Regardless of the composition ratio of MgO, the 3 days strength of the sample using water glass as the activator is less than the 3 days compressive strength of the samples using MS. However, the compressive strength of 7 and 28 days increased significantly, higher than that of MS. On the other hand, NaOH showed similar range of 3 days strength compared to MS, and the compressive strength of 7 and 28 days increased slightly compared to MS. Compared with NaOH, water glass had a smaller compressive strength at early-age strength (3 days) but a sharp increase in strength after 7 days.

The compressive strength values of alkaline activated slag cement using the same mixed activation of 5% NaOH + 5% Na_2_SiO_3_ and 10% NaOH + 10% Na_2_SiO_3_ were measured higher than MS [28]. Both 5% and 10% activators showed higher strength values than MS, and 10% activator showed better compressive strength value than 5%. This is consistent with the findings from previous studies on AASC that increasing the activator concentration increases compressive strength. In particular, mixed activators of sodium hydroxide and sodium silicate show a higher strength-improving effect than the use of each separately.

Comparing the mechanical properties of MS with other types of activators shows that MS has relatively low slag activation effect. In the previous study on AASC, high compressive strength was obtained from experiments in which sodium silicate was mixed with NaOH and KOH at concentrations of 6, 8 and 10 moL [23]. In general, it has been reported that the use of a mixture of sodium silicate and hydroxide-based activation can achieve high compressive strength. In other studies where 1% Na_2_O equivalent of Na_2_SO_4_ was used, an increased strength with age was also reported [18]. In an AASC study in which gypsum was used at 2% and 4% by slag mass [19], the compressive strength increased with the amount of gypsum. As reported by Wang et al. [29], the early compressive strength of AAS mortars activated with sodium sulfate is lower than that with other activators such as Na_2_CO_3_, NaOH and water-glass (sodium silicate). The recorded compressive strengths of mortars activated with 2 M Na_2_SO_4_ are 1.2, 5.1, 10.2 and 20 MPa at the ages of 1, 3, 7 and 28 days, respectively. These compressive strengths were lower than those obtained with other activators, particularly the early-age strength. In previous studies, a relatively low initial strength of AASC has been reported when using a sulfate as the activator [15,18,21,25].

The strength of AASC using sulfate as an activator showed relatively low initial and late strengths as compared with AASC using sodium hydroxide or sodium silicate as an activator. These low strength properties were also confirmed in this study using MS.

### 3.2. Hydration Products

**Figure 2:**
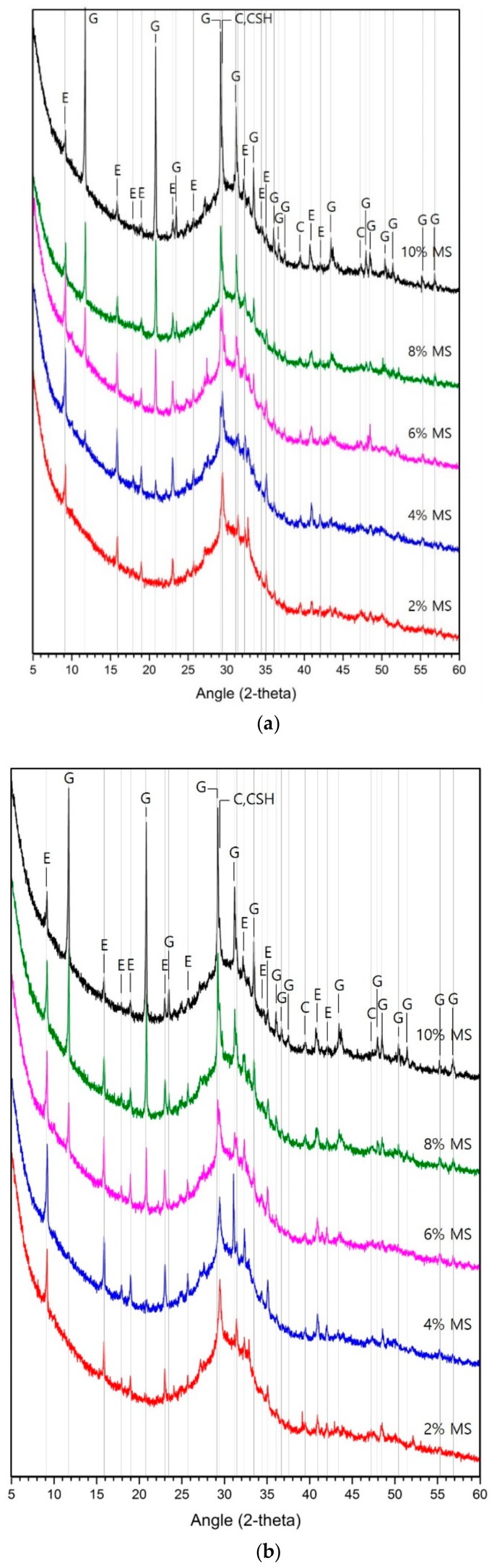

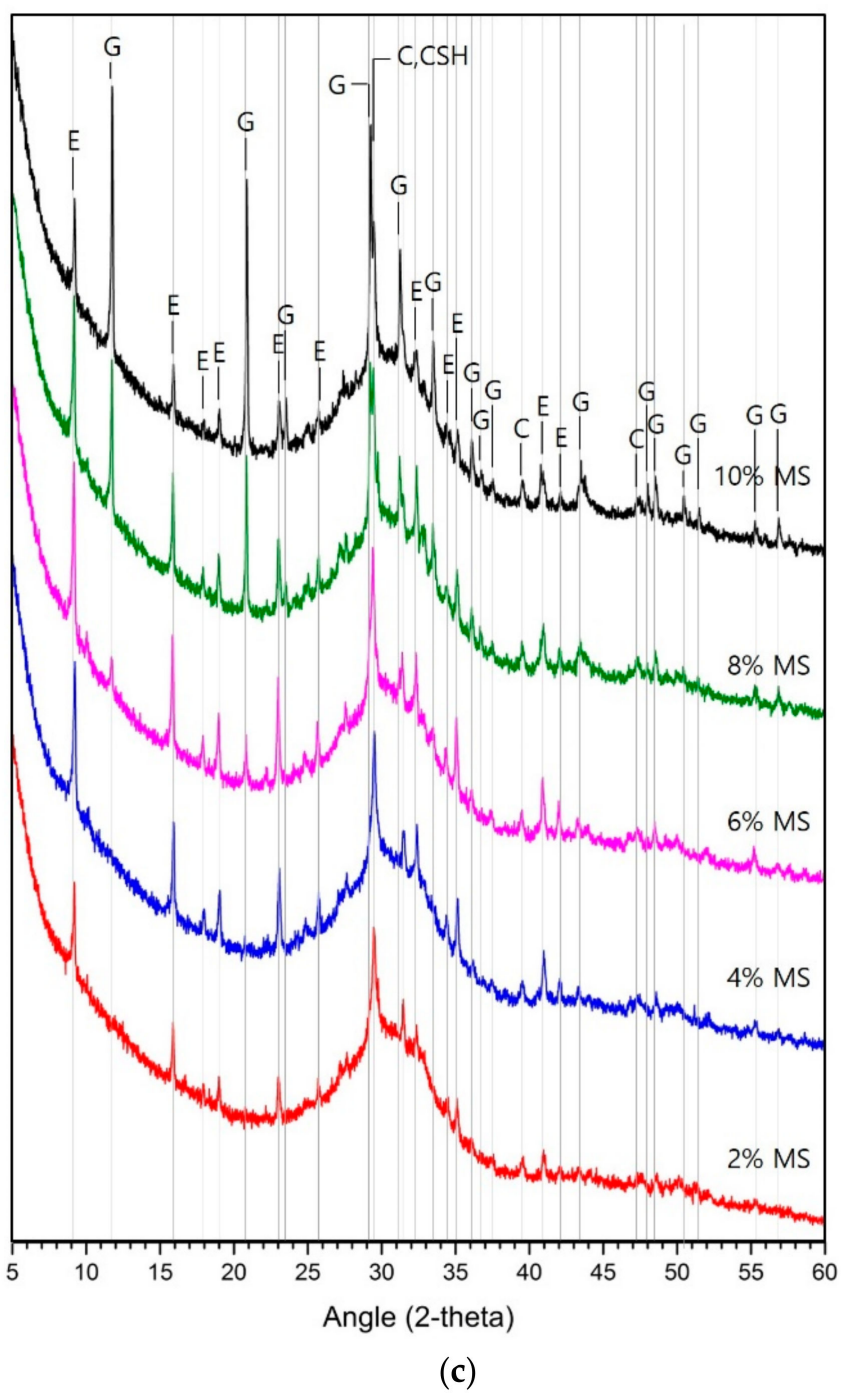
XRD analysis: (**a**) 3 days, (**b**) 7 days, (**c**) 28 days, E: Ettringite, G: Gypsum, C: Calcite, CSH: C-S-H gel, M: Magnesium oxide.

Figure 2 shows the results of XRD analysis according to the MS content for each age. The representative reaction products were ettringite, gypsum, calcite and calcium silicate hydrate (C-S-H). This is consistent with the reactants shown in AASC studies where Na_2_SO_4_, CaSO_4_ or gypsum (CaSO_4_·2H_2_O) was used as the activator [15,22,30].

As age increases, the ettringite and C-S-H peaks increase and the gypsum peak decreases. Ettringite and C-S-H are representative hydration products of AASC using sulfate as an activator. Therefore, the increase of ettringite and C-S-H peak means the hydration of GGBFS. From the compressive strength results shown in Figure 1 and the XRD results shown in Figure 2, the MS affects the hydration of GGBFS, but its reactivity can be assumed to be slow. However, as the concentration of MS increases, the C-S-H gel peak decreases slowly. Pan et al. [14] reported that as the SO_4_ increased, the C-S-H gel slowly disappeared and a significant amount of extensive ettringite was produced. Therefore, in this study using MS as an activator, the change of ettringite peak is clearly confirmed, but the change of C-S-H gel peak is not clear. In particular, as the concentration of MS increases, the C-S-H gel peak tends to decrease slightly. This is consistent with Pan et al. [14] report mentioned earlier. This slow hydration reactivity has already been reported in previous studies using sulfate such as Na_2_SO_4_ or CaSO_4_. Sahin et al. [25] showed that adding 10% gypsum to slag resulted in a high compressive strength when cured at 80 °C, but an unreacted-gypsum peak was still observed. In the present study using MS, a gypsum peak was observed even after 24 h at 80 °C.

The main reactant of slag activated with Na_2_SO_4_ (sodium sulfate) is C-S-H with a low Ca/Si ratio and a significant degree of aluminium substitution, referred to as C-A-S-H [15]. The main secondary reaction product has been identified as ettringite (3CaO·Al_2_O_3_·3CaSO_4_·32H_2_O) [15,16].

In previous studies on AASC using sulfate activators, it has been reported that the sulfate activator had an insufficient effect on the slag so that insufficient hydration of the GGBFS was achieved [15,21]. In the case of slag activated with Na_2_SO_4_, increasing the sulfate content increased the reactivity with the slag and led to a higher ettringite peak [15]. However, the results of the present study show that the ettringite peak tends to increase up to a certain MS content and then decrease.

In Figure 1, the maximum compressive strengths are found for 4% MS on day 3 [Figure 2a] and day 7 [Figure 2b] and with 6% MS on day 28 [Figure 2c]. In Figure 2, no gypsum peak is observed with up to 4% MS on days 3 and 7, and the highest ettringite peak is observed with 4% MS. However, from 6% to 10% MS, the gypsum peak increases sharply and the ettringite peak decreases sharply. At 28 d, no gypsum peak is observed until 6% MS, and the ettringite peak increases gradually, leading to the highest ettringite peak at 6% MS. The gypsum peak increases and the ettringite peak decreases from 8% to 10% MS. In Figure 2a, the gypsum peak is very small up to 4% MS. Portlandite and ettringite peaks were gradually increasing up to 4% MS. However, the gypsum peak was rapidly increased at 6% MS. Moreover, portlandite and ettringite peaks start to decrease from 6% MS. In Figure 2b,c, the MS content at which these changes occur is 4% and 6%, respectively. Therefore, the intensity of gypsum, portlandite and ettringite peaks change rapidly at 4% MS at 3 days, 4% MS at 7 days and 6% MS at 28 days. As shown in Figure 1, the highest compressive strength occurred at the MS content where the peak change was abrupt. From the XRD results, the maximum compressive strength is greatly affected by the increase in ettringite and the decrease in gypsum.

Therefore, as the contents of MS increases, the amount of gypsum increases. The gypsum activates GGBFS and forms ettringite. Gypsum is a major source of sulfate ions. Activation of GGBFS elutes Ca^2+^ and Al^3+^ ions from GGBFS. In slag experiments with 5% and 10% substitution of gypsum, the increase of gypsum contents increased the gypsum peak and ettringite peak in XRD analysis [22]. Therefore, gypsum affects the production of ettringite, but unhydrated gypsum is present between the hydration reactants, causing expansion and thus reducing compressive strength [22]. In contrast, in the study using sodium hydroxide or sodium silicate as individual admixtures or blending, the main hydration reactant was C-S-H gel [27,28]. In their XRD analysis, gypsum and ettringite were not observed. This may be due to the presence of sulfate (sodium sulfate, calcium sulfate, and magnesium sulfate), which is not present in sodium hydroxide or sodium silicate. As a result, the use of hydroxide or silicate series as an activator shows a rapid activation reaction in the initial hydration of slag. On the contrary, sulfate showed a slow activation reaction and major reactants were different.

This process is repeated and affects the change of hydration products and compressive strength. Unreacted-MS was not found in samples exceeding the MS content where the highest compressive strength occurred. This may be because MS decomposes into gypsum and magnesium compounds in the hydration reaction stage. This phenomenon is similar to that reported by Mobasher et al. [15], who detected no unreacted Na_2_SO_4_ in AASC activated with Na_2_SO_4_. The XRD analysis in Figure 2, no hydration reaction containing magnesium was observed. Presumably, it is believed to exist as a hydrated reactant in amorphous form not measured by XRD.

MS is separated into Mg^2+^ and SO_4_^2−^ by mixing with the mixture. SO_4_^2−^ activates slag particles to promote the elution of Ca^2+^ ions to form gypsum. Gypsum forms ettringite from Ca^2+^ and Al^2+^ ions eluted from slag. From this reaction up to 4% MS contents, most of the gypsum is consumed to form ettringite (3CaO·Al_2_O_3_·3CaSO_4_·32H_2_O). Therefore, in the XRD analysis of Figure 2, the gypsum peak was hardly observed until 4% MS contents. However, from contents exceeding 6% MS, more gypsum is formed than gypsum needed to form ettringite. As a result, excessively formed gypsum expands over time, increasing porosity and decreasing compressive strength.

### 3.3. Pore Structure

**Figure 3:**
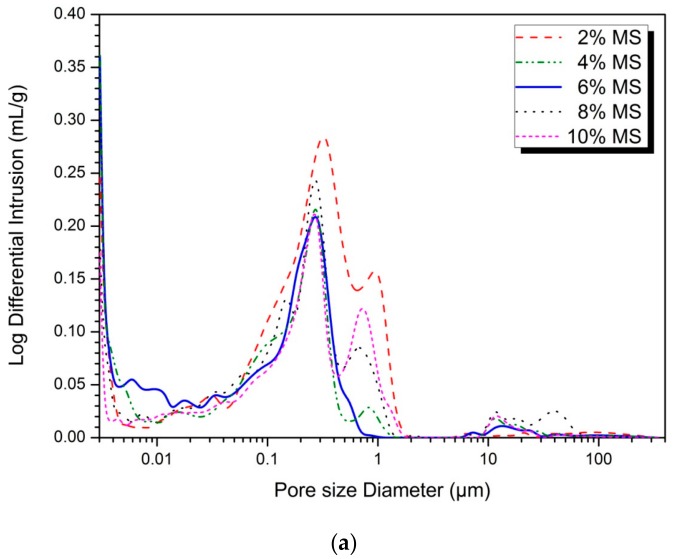

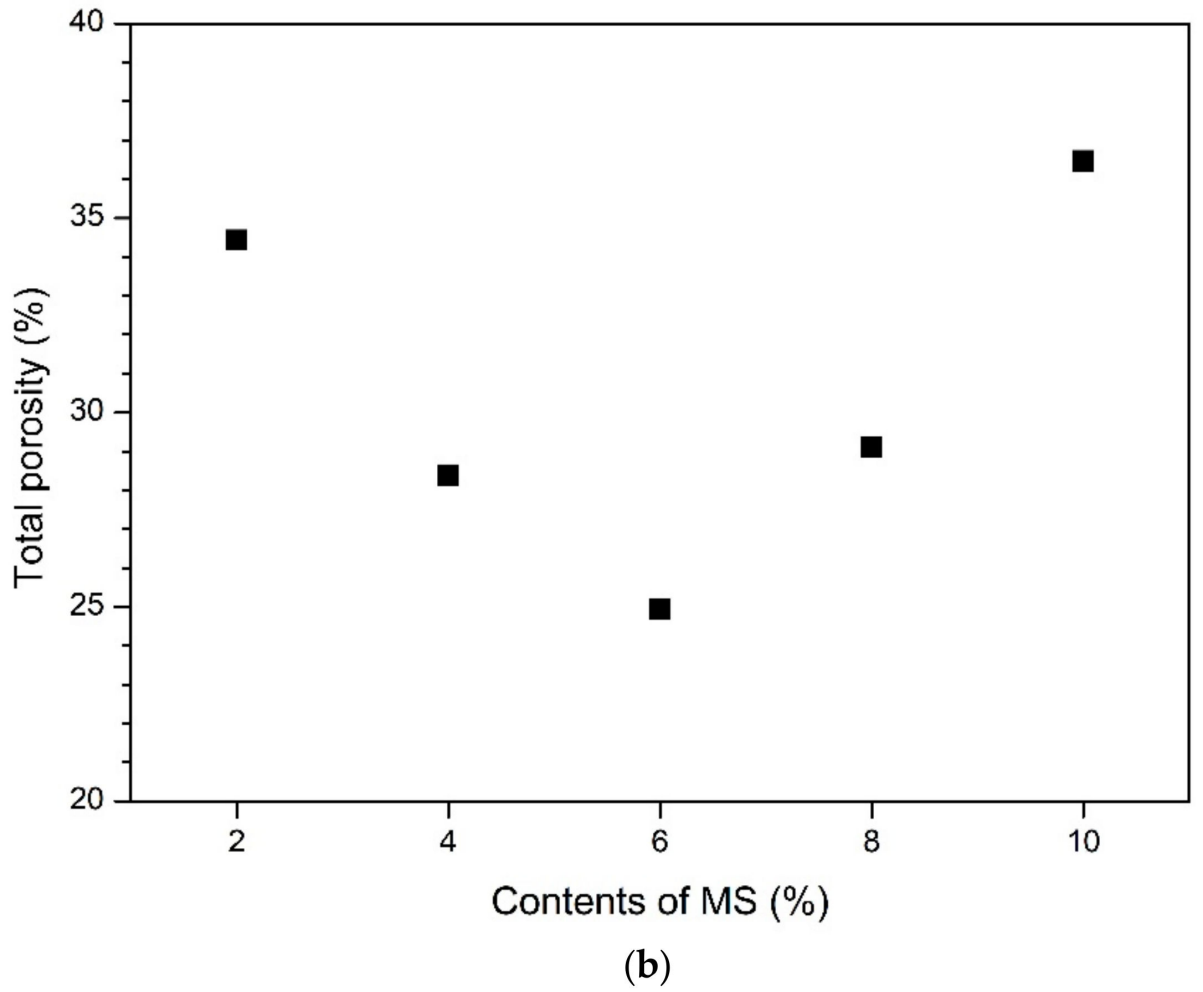
Pore structures at 28 days samples: (**a**) pore size distribution, (**b**) total porosity.

Figure 3 shows the pore size distributions and total porosities for different MS contents measured using MIP at 28 days. Although the true pore size distributions of the specimens might differ from the MIP results, as discussed in previous work [31,32], the MIP results are still useful for comparing the pore size distributions between the specimens.

Compared with the 6% MS sample with the highest intensity in Figure 3a, the remaining MS concentration samples show an increase in pores between 0.3 and 2.0 μm. The pores in the range of 0.3–2.0 μm are decreasing gradually in order of 2% > 10% > 8% > 4% > 6% MS. It is also observed that pores of 10–100 μm increase at MS concentrations other than 6% MS sample. The 6% MS sample showed an increase in pore size of 0.01 μm or less. In the compressive strength results of Figure 1, the magnitude of 28 days compressive strength increases in order of 10% < 2% < 8% < 4% < 6%. This increase in compressive strength is similar to the decreasing order of pore volume from 0.3 to 2.0 μm. Therefore, the amount of pores between 0.3–2.0 μm and 10–100 μm affects the change of compressive strength. The gypsum and ettringite shown in Figure 2 are the factors influencing the size change of pore size. Park et al. [22] reported that gypsum mixed with slag removed large pores (100 μm) and 0.1 μm pores because of the formation of fine ettringite and increased the strength by increasing 0.03–0.40 μm pores. The addition of excess gypsum also suggests that a large number of pores near 1 μm and pores with a size of 10–100 μm are generated to reduce the strength. This may be due to an expansion crack or a large pore between the relatively large re-precipitated gypsum crystals.

Figure 3b shows the total porosity of Park et al. [22] and Kim et al. [28] at 28 days. Park et al. [22] noted that the 10% gypsum sample had the lowest total porosity and the highest compressive strength. The 15% gypsum sample showed lower total porosity than the 0% gypsum sample. The cause of this result was reported that excessive gypsum caused expansion inside the specimen, increasing porosity and decreasing compressive strength. A study by Kim et al. [28] using 5% NaOH + 5% Na_2_SiO_3_ and 10% NaOH + 10% Na_2_SiO_3_ showed a sharp decrease in the total porosity of the 10% activator than 5%. Increasing the concentration of the activator promotes the formation of the hydration reactant and creates a dense structure, reducing the porosity. However, the sulfate-based activator is relatively slow in the hydration of the slag particles, delaying the formation of dense hydration reactants. As a result, the total porosity is higher than AASC using NaOH or Na_2_SiO_3_. However, since the factors affecting the total porosity are various, it is difficult to identify the clear tendency simply by the type and concentration of the activator. In recent studies, however, Na_2_SiO_3_ was found to have a more effective pore filling effect than NaOH [33]. Analysis of pore structures by activators requires much further research.

Table 2 shows the distribution according to pore size smaller than 10 μm [34]. The percentage of large-capillary pores decreases from 2% to 6% MS and increases from 8% to 10% MS. The percentages of medium-capillary pores and gel pores decrease from 8% to 10% MS after increasing from 2% to 6% MS. The 6% MS sample with the maximum compressive strength has the smallest percentage of large-capillary pores and the highest percentage of medium-capillary and gel pores. It is generally known that the presence of relatively large pores in a solid reduces its strength [35]. The correlation between the strength results and the pore size analysis of Figure 1 can be confirmed. The 10% MS samples with the lowest compressive strength showed the largest amount of large capillary pores and the least amount of gel pores. In contrast, the 6% MS sample has the smallest amount of large capillary pores and the largest amount of gel pores. The compressive strength was improved as the number of large capillary pores decreased and the number of gel pores increased. Therefore, it can be confirmed that MS affects the pore structure and compressive strength of the hydration reaction product. MS-activated AASC has an optimal amount of MS that produces the highest compressive strength.

### 3.4. Thermal Analysis

**Figure 4:**
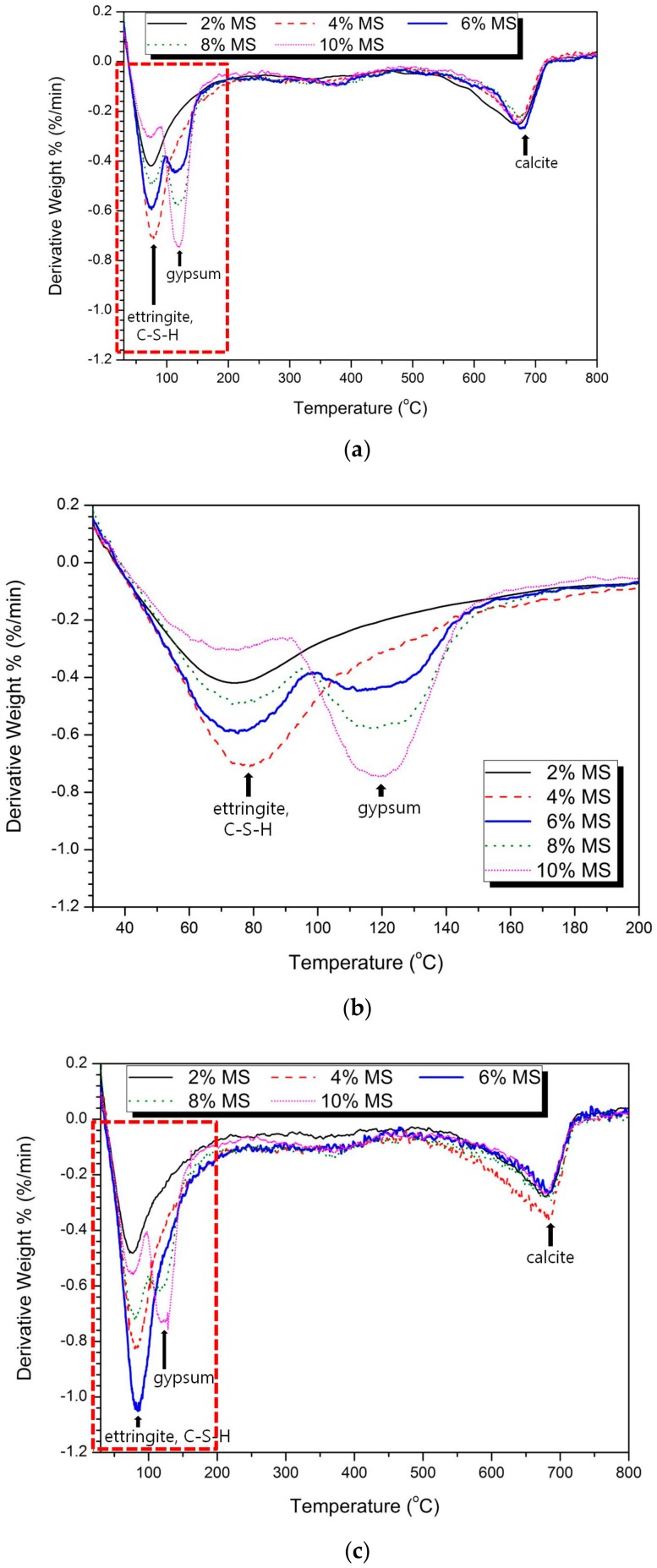

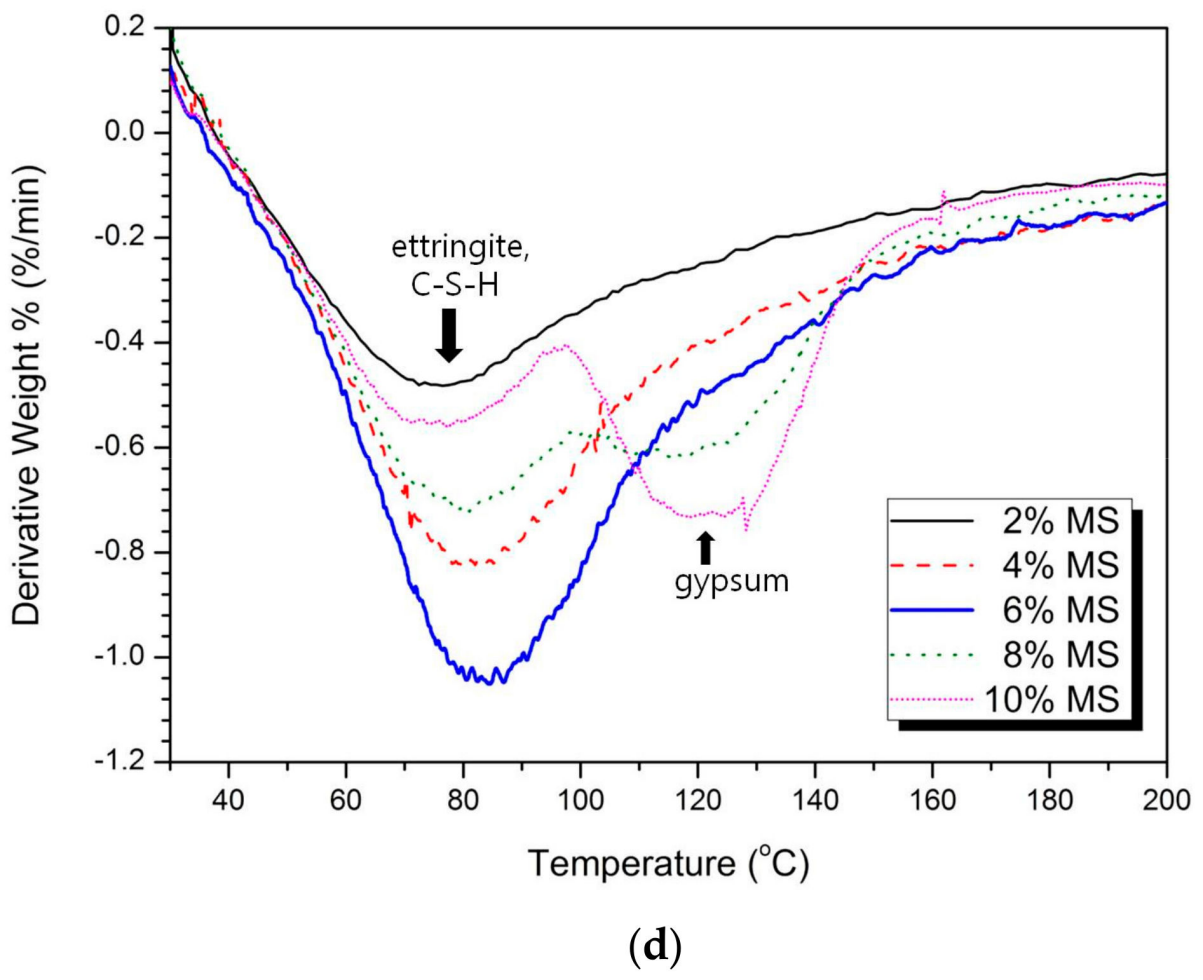
Thermal analysis results: (**a**) 3 days, (**b**) magnification of the red dotted rectangle in (**a**), (**c**) 28 days, (**d**) magnification of the red dotted rectangle in (**c**).

Figure 4 shows the thermal analysis (TG/DTG) results for the 3 d and 28 d samples. The TG/DTG analysis shows that the main reaction materials were ettringite and gypsum. These reactants correspond to the XRD results shown in Figure 2.

In Figure 4a,b, weight loss due to water loss of ettringite, C-S-H and calcite can be confirmed. The ettringite shows weight loss at 80 °C–130 °C [36] or 110 °C–150 °C [37]; C-S-H shows weight loss at 50 °C–200 °C [38] or 90 °C–110 °C [39], and gypsum shows weight loss at 80 °C–220 °C [40] or 100 °C–200 °C [41]. Calcite can be checked for weight loss at 665 °C–800 °C [41]. Due to the fact that the typical temperature ranges for the decomposition of C-S-H, ettringite and gypsum are narrowly located, it is difficult to precisely estimate the amounts of these reaction products [30]. However, because the amounts of C-S-H are similar between the samples (as concluded previously in the XRD analysis) and because the gypsum peaks near 144 °C are relatively small, the large peaks before 200 °C in Figure 4 are attributed mainly to the formation of ettringite.

Figure 4b,d show a large peak at 50–200 °C in the DTG curve because of the decomposition of ettringite and the loss of the adsorbed/inter-layer water of C-S-H. In Figure 4b, ettringite increases from 2% to 4% MS, with 4% MS yielding the maximum weight loss. However, the ettringite weight loss curve decreases sharply from 6% to 10% MS. Gypsum increases gradually from 2% to 10% MS with the highest weight loss. As shown in Figure 2a, the largest ettringite peak is observed with 4% MS with the highest compressive strength, and the gypsum peak is hardly observed. However, from 6% to 10% MS, the ettringite peak decreases and the gypsum peak increases sharply. The results of the thermal analysis are also consistent with the tendency of the hydration products in the XRD.

In Figure 4d, ettringite shows the maximum value with increasing weight loss until 6% MS and then decreases again to 10% MS. Gypsum increases gradually from 2% MS to the maximum weight loss at 10% MS. As shown in the XRD results in Figure 2c, the largest ettringite peak is observed at 6% MS with the highest intensity, and the gypsum peak is hardly observed. However, from 8% to 10% MS, the ettringite peak decreases and the gypsum peak increases steeply. No untreated MS, brucite (Mg(OH)_2_) or M-S-H gel was observed by thermal analysis.

### 3.5. pH Values

**Figure 5:**
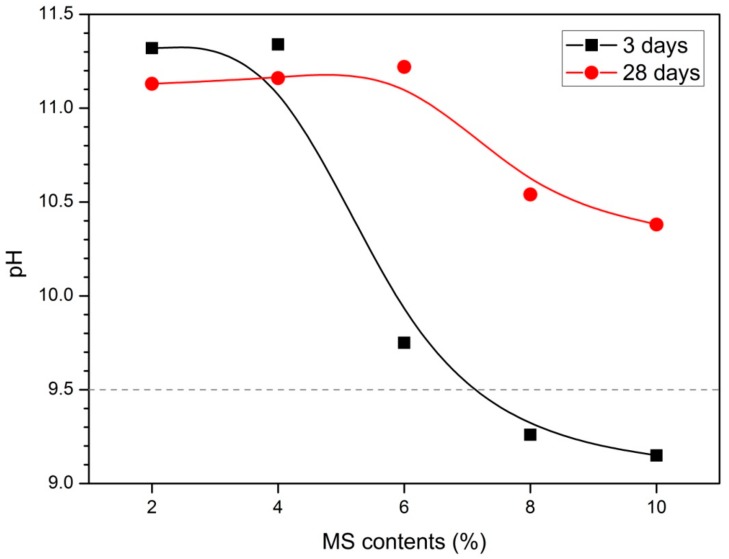
pH values for paste.

Figure 5 shows the results of pH measurements at 3 and 28 days. The 3 days samples had the highest value of pH 11.34 at 4% MS, and the 28 days samples had the highest value of pH 11.22 at 6% MS. The highest compressive strength was measured in the MS contents with the highest pH. This is clearly seen in comparison with the compressive strength results in Figure 1. At 3 days 0% to 4% MS was measured in the range of pH 11.0 to 11.5, but contents above 6% MS showed values less than pH 10.0. This decrease in pH value is clearly seen as the contents of MS increase. In particular, 6% MS shows a sharp decrease in pH compared to 4% MS. At 28 days, pH values from 0% to 6% MS were measured in the 11.0–11.5 range. However, 8% and 10% MS were measured to be less than pH 11.0 and 10% MS showed the lowest pH value.

The pH of the activator plays an important role in the hydration of slag. Song et al. [42] suggested that a pH higher than 11.5 is required for the hydration of slag to be activated effectively. It was also reported that the slag was not hydrated at pH lower than 9.5. Puertas et al. [43] noted that the activation process is delayed at low pH below 12. In addition, previous studies have reported that C-S-H gels do not form in environments with pH lower than 9.5 [42,43,44]. Therefore, at low pH (<9.5), a dense matrix is not formed, which affects high porosity and low compressive strength. All samples in this study at 3 and 28 days showed pH values below 12. In particular, pH values of less than 9.5 were measured for 8% and 10% MS at the 3 days sample. The pH lower than 11.5 causes the compressive strength to be low due to insufficient activation of slag. The hydration delay of slag affects not only the initial compressive strength but also the late compressive strength at 28 days. Although 6% MS at 28 days has the highest intensity value, it shows below 25 MPa. This is lower than the compressive strength results of activated slag experiments using sodium sulfate of Rashad et al. [21] as an activator and cured at 40 °C. In the results of Rashad et al. [21], pH was observed between 12.0 and 12.5 regardless of slag fineness. Compared with the experiments of Rashad et al. [21], this study showed a low pH despite higher 24 h curing temperature (80 °C) and higher MS contents (~10%). MS is thought to have lower activation capacity than sodium sulfate.

Low activation of MS delays hydration of slag. Therefore, other methods may need to be added along with MS to improve the activation of slag. For example, the addition of activators, increased concentrations, higher curing temperatures and mixing of different admixtures should be considered.

### 3.6. Microstructure

**Figure 6:**
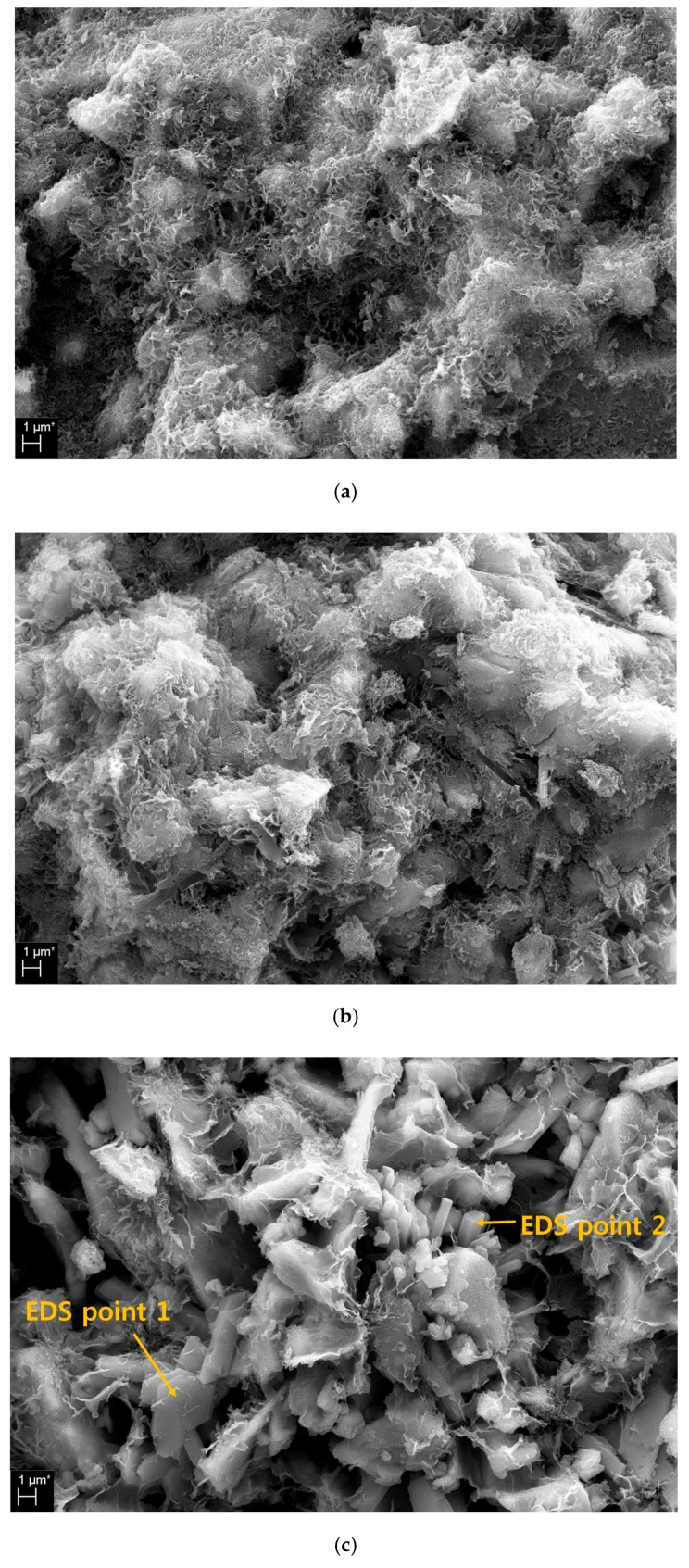
SEM images: (**a**) 2% MS, (**b**) 6% MS, (**c**) 10% MS.

Figure 6 shows SEM images of the 28 d samples with 2%, 6% and 10% MS. Figure 6a,b shows no significant difference in the SEM images of the 2% and 6% MS samples. The pores are visible and can be seen covered with hydration products. However, the 10% MS sample shown in Figure 6c shows a very rough surface, unlike the 2% and 6% MS samples. There are a number of pores, unreacted GGBFS particles and some hydration products on the observed surface.

In particular, EDS analysis was performed for ‘EDS point 1’ and ‘EDS point 2’ in the 10% MS sample. Table 3 shows the results of EDS analysis for EDS points 1 and 2 in Figure 6. ‘EDS point 1’ is a polygonal plate type and EDS analysis result is gypsum. ‘EDS point 2’ is identified as ettringite by EDS analysis with a short bar shape.

In Figure 1, 10% MS with the lowest compressive strength values was able to identify multiple pores, few hydration products and gypsum through SEM image observation. The SEM images show that the coarse crystals of gypsum or ettringite produce porous matrices because of the excessive addition of MS. This is similar to a previous study in which excess gypsum concentration formed a coarse porous matrix by gypsum or ettringite [22].

However, at the optimum MS concentration, strength enhancement by ettringite and gypsum can be obtained. The growth of ettringite and/or gypsum might cause two opposite effects for the strength development at an early reaction age. First, when ettringite and/or gypsum crystals grow appropriately in the pore spaces, this crystal growth might result in a pore-size-refining effect that promotes strength development. Second, however, if these crystals grow too much in the pores at an early reaction age, the overall matrix could expand and cause micro-cracks, which are probably the micro-cracks or pores seen in Figure 6c [22]. Therefore, the low compressive strength with 10% MS can be explained by the abundant presence of gypsum and the scarce presence of ettringite noted in the XRD and TG/DTG analyses. As noted in the MIP results, a high content of MS increases the number of pores around 1 μm and 10–100 μm. This can be explained by the excessive expansion of gypsum or ettringite as described above.

**Figure 7:**
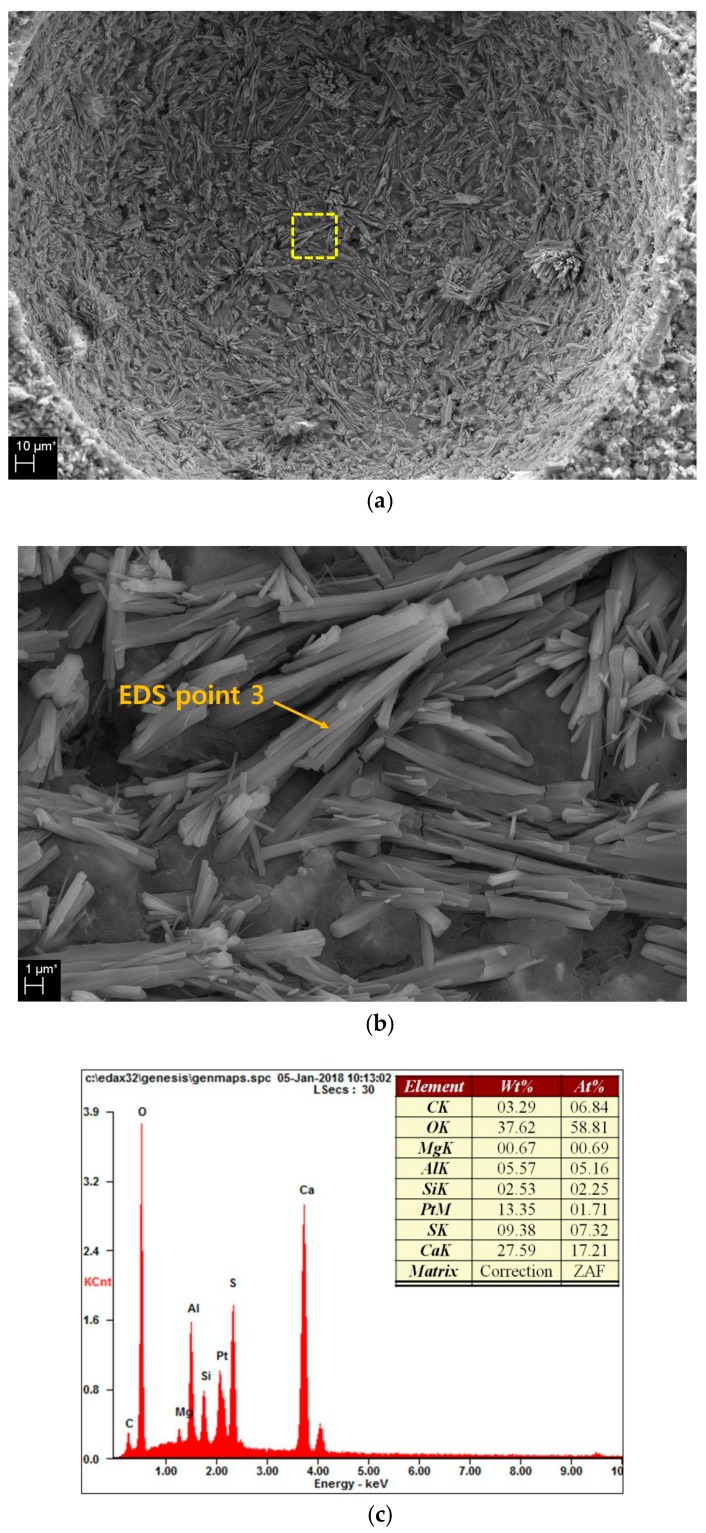
SEM in pore: (**a**) pore in 6% MS specimens, (**b**) magnification of hydration production, (**c**) EDS result of “EDS point 3”.

Figure 7a shows the hydration reaction material generated in the pores of the 6% MS sample. Numerous hydration reaction materials are generated inside the pores. Figure 7b shows an enlargement of the yellow dotted rectangle in Figure 7a, wherein a long bar-shaped material can be observed. An EDS analysis of one of these materials (‘EDS point 3′) is shown in Figure 7c. The result is ettringite, which is the hydration reaction material noted in the XRD and TG/DTG analyses and is also the substance noted to decrease the pore size and amount in the MIP results.

## 4. Conclusions

The mechanical and microstructural characteristics of AAS paste using MS as the activator are summarised as follows.

The MS concentration that yielded the highest compressive strength differed according to age. The highest strength occurred with 4% MS at 3 and 7 days and with 6% MS at 28 days. This means that as the age increases, the activation of GGBFS by MS occurs slowly. In addition, the low early strength is similar to those found in previous studies of AAS using sulfate activators. Therefore, additional studies are required to improve the initial strength of AAS using MS.

Through XRD and TG/DTG analyses, the typical hydration reaction materials were found to be gypsum and ettringite/magnesium oxide. At the highest MS concentration, the highest ettringite peak was observed, and the gypsum peak was not observed. In addition, the maximum strength was observed with the MS concentration for which the weight loss of ettringite was the largest, and the lowest intensity, namely, that with 10% MS, showed the lowest ettringite peak and the highest gypsum peak and weight loss rate. Therefore, it was confirmed that ettringite and gypsum are hydration reaction materials that have a significant effect on the increase of compressive strength.

The pore structure characteristics were confirmed by MIP analysis. The lowest percentage of large-capillary pores and highest percentages of medium-capillary and gel pores was shown by 6% MS with the highest strength. This is due to the effect of pore structure reduction by the pore-filling action of ettringite. However, 10% MS showed the largest percentage of large-capillary pores and the smallest percentages of medium-capillary and gel pores. This is the result of excess gypsum swelling due to excessive MS concentration.

SEM images showed the hydration products in the 2%, 6% and 10% MS samples. The 2% and 6% MS samples showed similar reaction products and surface states. However, the 10% MS sample was found to have multiple pores, rough surface features, polygonal gypsum and long rod-shaped ettringite. Excessive gypsum formation and swelling of the gypsum due to excess MS concentration cause the matrix to generate many pores and cracks, thereby reducing the strength.

Slag using MS as an activator showed a pH value lower than 11.5 on both 3 and 28 days. This tendency becomes clearer with decreasing pH as the contents of MS increase. Therefore, MS has an alkaline environment lower than pH 11.5, which delays the hydration of slag. As a result, the matrix is not compact, and the compressive strength is lowered. Therefore, in order to use MS as the sole activator, it is necessary to use a combination with other alkali activators, increase the fineness of slag or use an additional admixture.

## Figures and Tables

**Table 1:** Chemical and physical properties of GGBFS.

	Chemical Components (%)								Density (g/cm^3^)	Fineness (m^2^/kg)	LOI (%)
	SiO_2_	Al_2_O	Fe_2_O	MgO	CaO	K_2_O	SO_3_	Na_2_O			
GGBFS	35.30	12.58	0.79	3.19	41.30	0.63	4.75	0.33	2.84	420	0.82

**Table 2:** Analysis of pore sizes.

Contents of MS	Large Capillary Pores (10–0.05 μm, %)	Medium Capillary Pores (0.05–0.01 μm, %)	Gel Pores (<0.01 μm, %)
2% MS	75.71	6.93	17.36
4% MS	55.19	8.38	36.43
6% MS	51.10	11.97	36.93
8% MS	68.85	10.59	20.56
10% MS	73.02	10.55	16.43

**Table 3:** Results of EDS analysis (Atomic %).

	Mg	Al	Si	S	Ca
EDS point 1	1.55	1.74	4.27	15.46	19.65
EDS point 2	3.99	5.62	12.36	0.95	12.98
